# Assessing the augmentation of *Amblydromalus limonicus* with the supplementation of pollen, thread, and substrates to combat greenhouse whitefly populations

**DOI:** 10.1038/s41598-018-30018-3

**Published:** 2018-08-15

**Authors:** Ming Hui Lee, Zhi-Qiang Zhang

**Affiliations:** 1 School of Biological Sciences, The University of Auckland, Auckland, New Zealand; 2 Landcare Research, Auckland, New Zealand

## Abstract

Due to issues with establishment and persistence of natural enemies in biological control, the provision of alternative food sources and oviposition sites are important factors to enhance pest control. In this study, three different supplementation treatments were examined for their ability to increase the populations of the predatory mite *Amblydromalus limonicus*, and its implications for greenhouse whitefly control on peppers and eggplants. These were: (1) pollen (*Typha orientalis*), (2) pollen and thread, (3) pollen, thread, and a substrate mixture of buckwheat, gorse, and rice husks, which were compared to a control treatment that had no supplementation. Significant treatment effects were found on pepper for *A*. *limonicus* (mite eggs *p* = 0.008, mobile mites *p* = <0.0001). The predatory mite successfully established and persisted at high population levels in the pollen-thread, and pollen-thread-substrate treatments. All supplementation treatments were able to control whitefly populations on peppers, while the control treatment failed to. The results obtained were formulated into possible application techniques for greenhouse growers to utilise.

## Introduction

**Figure 1:**
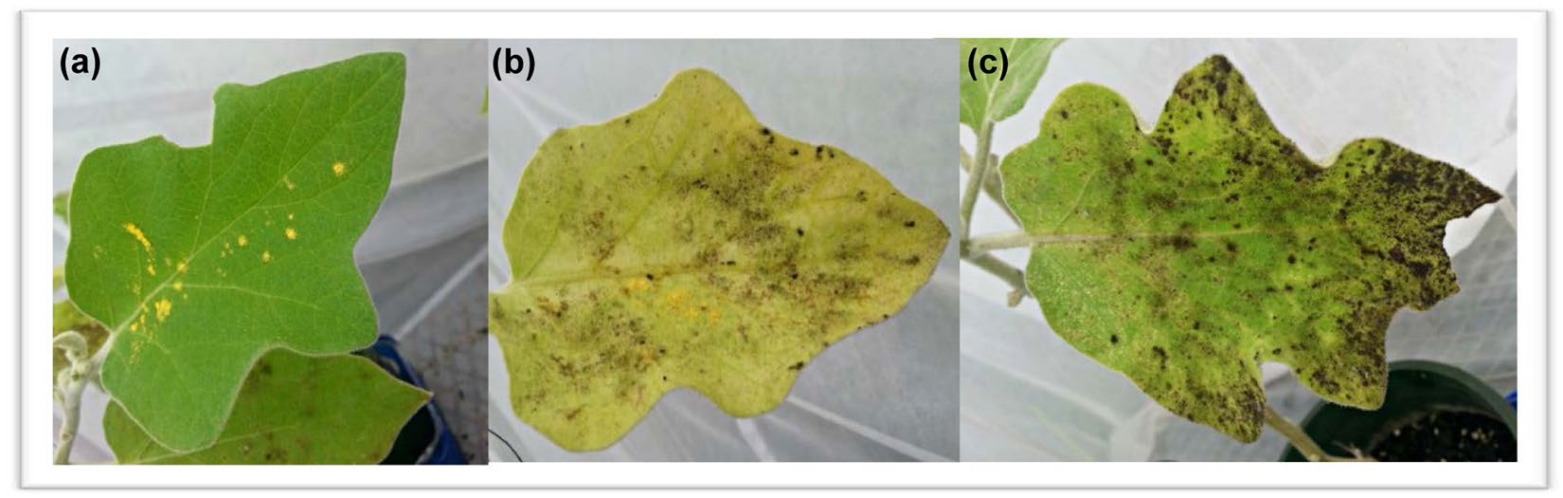
A healthy eggplant leaf with pollen supplementation for the predatory mite, *Amblydromalus limonicus* (**a**) compared to a yellowing leaf from greenhouse whitefly infestation (**b**), and a leaf with black sooty mould fungi (*Capnodium spp*., Mont) colonising the vast amounts of honeydew excreted by greenhouse whitefly (**c**).

Greenhouse whitefly (*Trialeurodes vaporariorum*, Westwood) is one of the world’s most invasive greenhouse pests attacking over 800 species of host plants belonging to over 450 genera^1,2^. These hosts include a range of economically important agricultural and ornamentals plants such as cucumber, eggplant, tomato, pepper, poinsettia and chrysanthemum^3^. This wide host range leads to serious horticultural issues where the surrounding greenhouse climate is highly favourable for whitefly development. Whiteflies cause indirect damage to agricultural crops via their honeydew secretions which are commonly colonised by a black sooty mould fungi^4^. This effectively blackens the leaf and fruit surfaces thereby inhibiting photosynthesis, leading to economic losses of fruit and ornamental crops^1,5,6^ (Fig. 1). Furthermore, due to the piercing and sucking mouthparts of *T*. *vaporariorum*, they are capable of transmitting disease-causing plant viruses; however, this is not reported to be an issue within New Zealand^3,7^ Each female *T*. *vaporariorum* is able to produce an average of 320 eggs within their life cycle^8^. In warm conditions, such as in greenhouses, whiteflies are able to continue this high reproduction rate throughout the year^2,7^. High reproductive capacity and lack of a dormant state means that greenhouse whitefly are able to reach and sustain high pest levels within a few generations. The compounded effects of both direct and indirect damage can result in defoliation, stunted growth, reduced yield and even death which demonstrates the enormity of the potential economic damage that they pose^7,9^.

Growers in New Zealand currently use a combination of control methods for greenhouse whitefly. This includes the biological control agent *Encarsia formosa* (Gahan), an entomopathogenic fungi (Biotelliga products), and the use of chemical control^5^. Unfortunately, these methods have not been able to provide sufficient control and greenhouse whitefly remains an important pest species within greenhouses in New Zealand^5^. Accordingly, additional measures need to be investigated to combat this invasive pest.

Phytoseiid mites have been shown to be an effective biological control agent for a range of thrips, phytophagous mites, whiteflies, and other agricultural pests^10–14^. Not only can predatory mites reduce or eliminate pest populations, but the inexpensive rearing methods make them an excellent option for greenhouse growers^15–18^. In addition, they are a superior alternative to pesticides which have drastic consequences for human health and the environment^19–21^. *Amblydromalus limonicus* (Garman & McGregor) is a generalist predator which naturally occurs within New Zealand^10,22–25^. The predatory mite does not enter reproductive diapause in short day photoperiods during cool night temperatures which results in year round control^26^. In addition, a higher fecundity and shorter developmental times has also been observed when the predatory mite was reared on plant exudates and pollen, compared to artificial diets^27,28^. This absence of diapause and ability to exploit an array of food sources, strengthens its capability as a biological control agent.

*Amblydromalus limonicus* has been shown to outperform other predatory mites in the control of greenhouse whitefly on strawberries, cucumbers, and especially roses^10,12,29^. From these findings, it is safe to assume that *A*. *limonicus* is superior to *E*. *formosa* on cucumber plants where the parasitoid has been found to have great difficulty in controlling greenhouse whitefly populations^30^. However, similar to the parasitoid, the ability of *A*. *limonicus* to control pests is regulated by physical plant traits^31,32^. This suggests that the predator may not be suitable for control in crops that have leaves with dense glandular trichomes. Other limitations of *A*. *limonicus* in biological control operations are its cannibalistic behaviour and the sensitivity of its eggs to low humidity^26,33^.

In augmentative biological control programs, large numbers of predators are often released on crops in an attempt to reduce or eliminate pest populations^34^. Although this practice is widely adopted in agricultural systems, it is unlikely to replace broad-spectrum pesticides due to an apparent lack of efficacy^34–36^. Poor establishment and short term persistence of natural enemies are one of the main issues cited in biological control^36,37^. One possible method to overcome this problem is to routinely release predators, however this is not always an economically viable option for producers. Several strategies that have been adopted to increase the establishment and persistence of natural enemies include provision of alternative food, prey, hosts, oviposition sites, and shelter^35,36,38,39^. The supplementation of pollen has been shown to provide effective pest control through the numerical response of predators^38,40,41^. Substances such as twine have been beneficial through the provision of additional oviposition sites. Moreover, a study found that when both pollen and twine were supplied, predatory mite populations increased by more than tenfold due to the provision of alternative food and oviposition sites^39^. These studies indicate that both pollen and twine provisioning can assist in predatory mite establishment and serve to increase the efficacy of biological control programmes under greenhouse conditions.

The aim of this study was to investigate the ability of *A*. *limonicus* to control greenhouse whitefly populations on two different host plants. Pepper (*Capsicum annuum*, Linnaeus), or bell pepper, was chosen due to its glabrous leaves and its low preference by whitefly, while eggplant (*Solanum melongena*, Linnaeus) was selected due to its dense trichome coated leaves and high preference by whitefly^42^. Buckwheat, gorse, and rice husks were chosen as the substrate as they have shown to be more superior in reducing cannibalism than other tested substrates^43^. Three different treatments which had either a subset, or a combination of pollen, thread, and a mixture of the husks were assessed on their ability to optimise natural enemy populations. These treatments were compared to a control treatment which contained no pollen, thread, or rearing media. The substrate treatment was added in an attempt to increase the persistence and establishment success of *A*. *limonicus*. The addition of husks was to provide a reservoir where stages vulnerable to low humidity and cannibalism could reside. Other benefits include the provision of a more favourable microclimate and an enhancement in natural enemy movement^43–46^. Pollen was provided as an additional food source while the thread was used to provide additional oviposition sites. It was predicted that a higher level of control would be observed on pepper plants compared to eggplants due to its glabrous leaves. Additionally, treatments which included pollen were expected to yield higher populations of *A*. *limonicus* compared to the control treatment and hence result in lower population levels of *T*. *vaporariorum*. This study is one of the few that will directly assess if an increase in predator populations results in better pest control. These results will add to the literature surrounding the use of *A*. *limonicus* on different host plants as well as methods of increasing its applicability in biological control of whitefly.

## Result

### Host plant effects

An ANOVA test which separated out the two different host plants found that for peppers, the treatment effect was significant across all mite and whitefly life stages, with the exception of whitefly eggs (Table 1). This effect was not duplicated across any of the life stage variables on eggplants. The sampling week also had a very strong significant effect on both mite and whitefly populations for both peppers and eggplant (P < 0.0001).

**Table 1:** Effect of different supplementation treatments on the different life stages of the predatory mite *Amblydromalus limonicus* and the greenhouse pest *Trialeurodes vaporariorum*.

Source of variation	Life stage									
	Mite Egg		Mobile Mite		Whitefly Egg		Whitefly Juvenile		Whitefly Adult	
	*F*	*P*	*F*	*P*	*F*	*P*	*F*	*P*	*F*	*P*
Pepper										
Treatment	5	**0**.**008**	36.6	<**0**.**0001**	2.57	0.078	3.95	**0**.**02**	3.11	**0**.**045**
Week	14.04	<**0**.**0001**	37.8	<**0**.**0001**	4.66	<**0**.**0001**	7.82	<**0**.**0001**	13.94	<**0**.**0001**
Treatment × Week	1.92	**0**.**006**	6.55	<**0**.**0001**	2.0	**0**.**004**	2.43	**0**.**0002**	1.87	**0**.**008**
Eggplant										
Treatment	1.16	0.35	1.04	0.397	1.37	0.312	1.62	0.217	2.49	0.09
Week	7.87	<**0**.**0001**	19.2	<**0**.**0001**	41.18	<**0**.**0001**	78.7	<**0**.**0001**	96.19	<**0**.**0001**
Treatment × Week	1.05	0.401	0.85	0.685	0.74	0.822	0.65	0.903	0.84	0.693

Shown are the results from a repeated measures ANOVA conducted on each host plant separately across 10 weeks of sampling. *F* refers to the F statistic produced from each ANOVA test. The degrees of freedom across all life stages for Treatment, Week, and Treatment × Week were 3, 9, and, 27 respectively.

### Sampling week interaction effect

No interaction effect was found between treatment and sampling week for eggplants (Table 1). However, for pepper plants, a strong significant interaction effect was found for all life stage variables in mites and whiteflies (Table 1). Thus, a further ANOVA test was employed to separate out each sampling week for pepper. The effect of different treatments was most notable across the different sampling weeks for mobile mites (Table 2). Treatment effects were primarily significant towards the end of the sampling period for the different whitefly life stages, with only one significant effect seen early on during week 1. This can be attributed to the small sustained population of whitefly that was obtained after additional releases.

**Table 2:** The effects of different treatments on the population dynamics of the different life stages of the predatory mite *Amblydromalus limonicus* and the greenhouse whitefly (*Trialeurodes vaporariorum*).

Week	Life stage									
	Mite Egg		Mobile Mite		Whitefly Egg		Whitefly Juvenile		Whitefly Adult	
	*F*	*P*	*F*	*P*	*F*	*P*	*F*	*P*	*F*	*P*
1	1.39	0.27	0.73	0.547	0.24	0.869	1	0.41	3.85	**0**.**022**
2	11.29	**0**.**0001**	7.36	**0**.**001**	0.18	0.911	1.69	0.196	0.22	0.882
3	1.31	0.293	5.74	**0**.**006**	1.13	0.358	1.5	0.24	0.35	0.786
4	2.09	0.128	12.35	<**0**.**0001**	0.15	0.929	1	0.41	0.39	0.759
5	0.33	0.802	16.87	<**0**.**0001**	0.20	0.898	1	0.41	1.64	0.207
6	0.39	0.76	33.76	<**0**.**0001**	0.59	0.627	1	0.41	0.239	0.869
7	1.19	0.334	21.82	<**0**.**0001**	9.32	**0**.**0003**	1	0.41	3.43	**0**.**033**
8	4.36	**0**.**014**	13.94	<**0**.**0001**	4.23	**0**.**016**	3.87	**0**.**022**	1.01	0.404
9	1.51	0.237	10.4	<**0**.**0001**	3.21	**0**.**041**	2.34	**0**.**049**	5.76	**0**.**004**
10	2.17	0.117	18.33	<**0**.**0001**	2.59	0.076	3.827	**0**.**024**	5.63	**0**.**005**

Shown are the results from a multistrata ANOVA model conducted on the different life stages found each week on pepper plants. *F* refers to the F statistic produced from each ANOVA test. The degrees of freedom across all life stages for each week were 3.

### Stem and Temperature

Overall, the largest diameter changes over the 10 week period were observed on pepper plants. The highest stem diameter increase was seen in the treatment containing pollen, thread, and substrate (PTS, PT = pollen and thread). However, there was no significant treatment effect on the stem diameter difference for either host plant (pepper: *F* = 1.73, *df* = 1, *p* = 0.20, eggplant: *F* = 0.173, *df* = 1, *p* = 0.682).

A significant difference was found in the temperatures between the two different greenhouse rooms (*F* = 87.89, *df* = 1, *p* = <0.001). The average temperature in the greenhouse room containing peppers was 20.3 °C (range: 11–33 °C), while for eggplants, the mean temperature was 18.9 °C (range: 10–35 °C).

### Treatment and host plant effects on population dynamics of *Amblydromalus limonicus* and *Trialeurodes vaporariorum*

**Figure 2:**
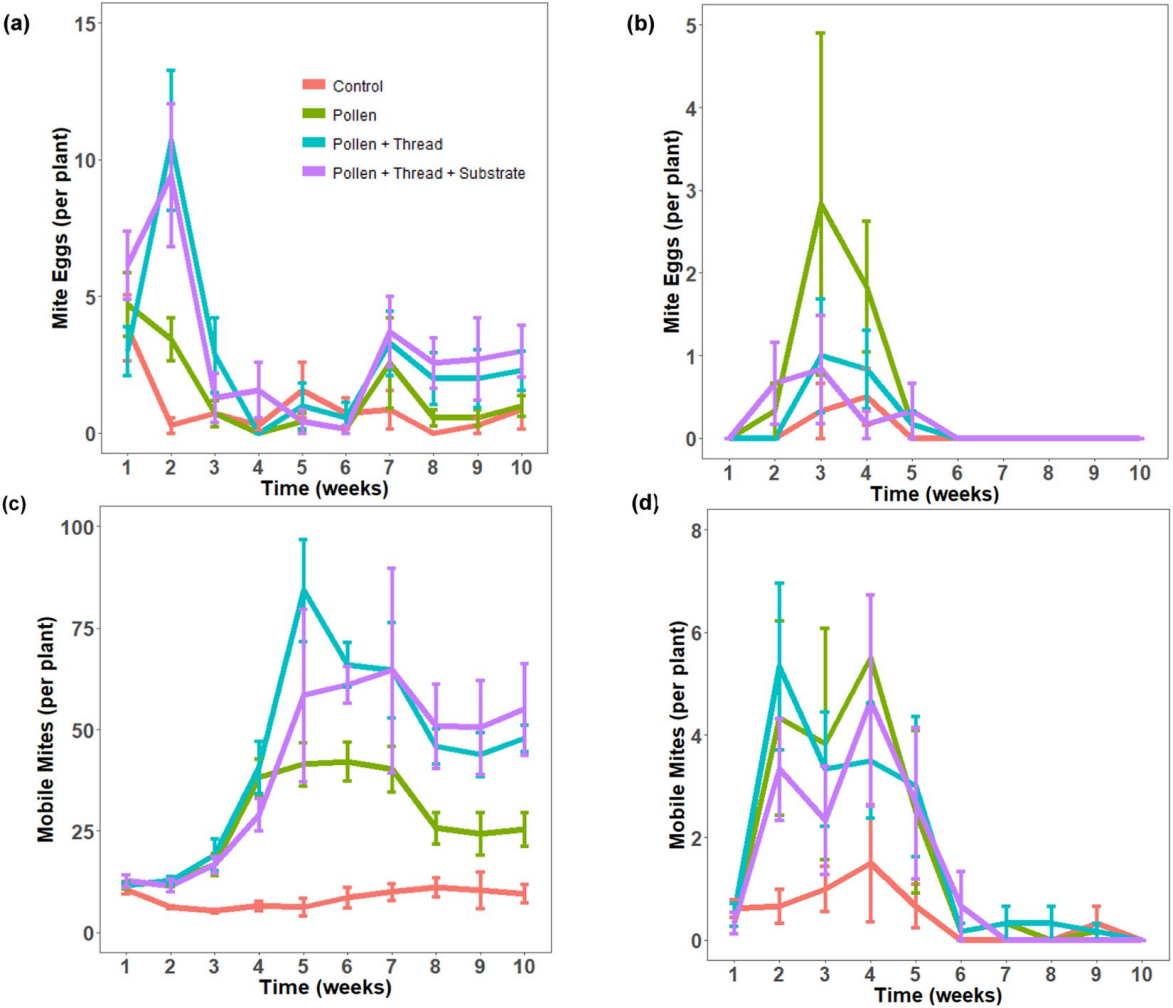
Comparison of the number of mite eggs and mobile mites on two host plants. The mean populations of eggs and mobile mites (nymphal and adult stages) of *Amblydromalus limonicus* for four different supplementation treatments on peppers (**a**,**c**), and eggplants (**b**,**d**). This experiment occurred in two different greenhouse rooms with variable temperature and humidity settings where the population dynamics were monitored weekly over a period of 10 weeks. Error bars are standard errors.

Although the two plant types were analysed in different greenhouse rooms, a large difference can be seen in the performance and population of predatory mites between the two host plants (Fig. 2). Differential treatment effects were prevalent for both mite eggs and mobile mites in pepper plants, however, this was not demonstrated in eggplants. In peppers, the PT and PTS treatments achieved the highest mite populations while the control performed the worst. For eggplants, very few mobile mites were detected throughout the experimental period with numbers drastically being reduced from week 6 onwards. Moreover, fewer mite eggs were discovered throughout the experimental period with no eggs found from week 6 onwards. It is also important to note the discrepancy in the abundance of predatory mites between the two host plants. Pepper plants hosted a much larger number of mite eggs and mobile mites compared to eggplants.

**Figure 3:**
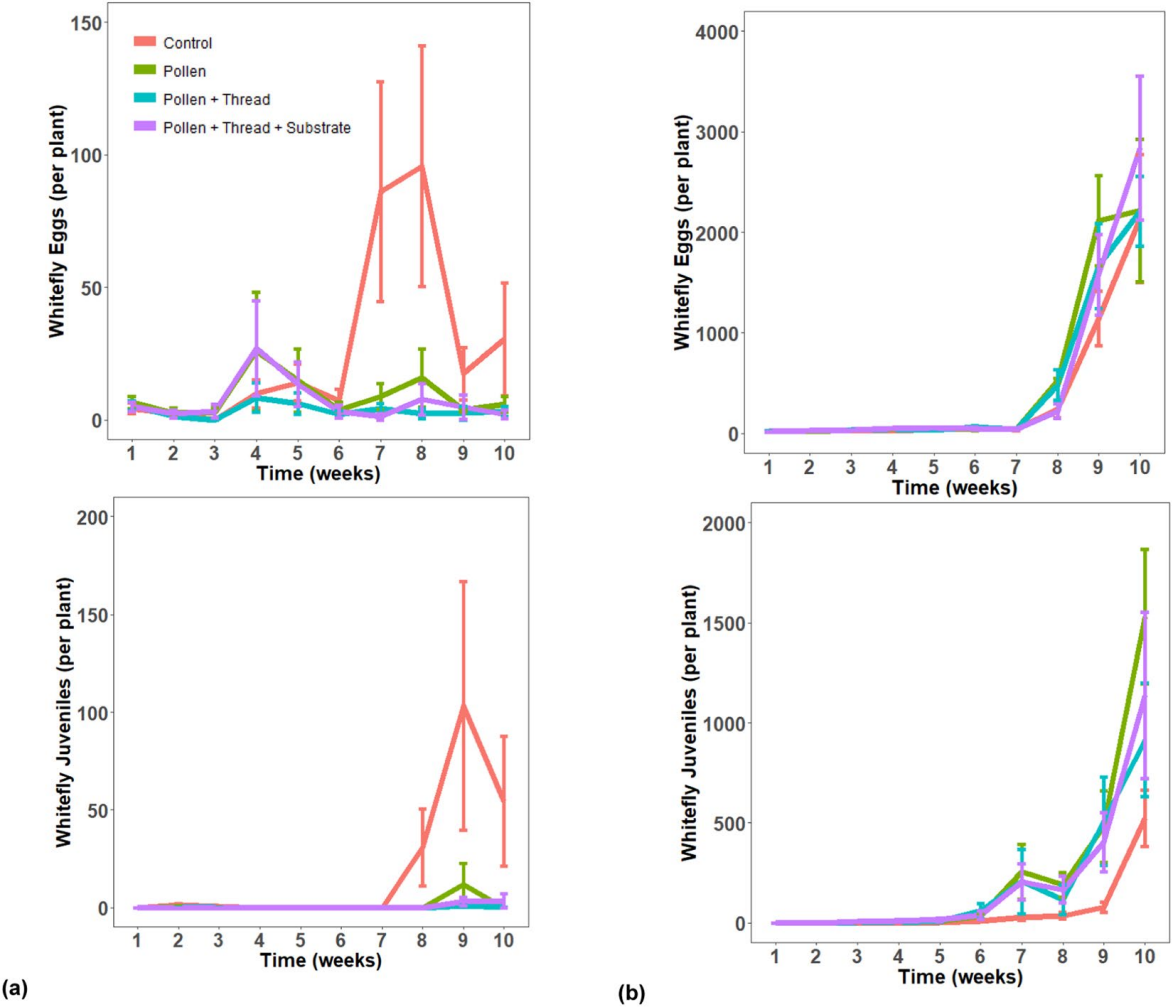
Comparison of the number of whitefly immatures on two host plants. Effects of four different pollen, thread, and substrate treatments on the mean density of eggs and juveniles (nymphal and pupal stages) of the greenhouse whitefly *Trialeurodes vaporariorum*. This experiment was conducted on two different host plants, pepper (**a**, **left**) and eggplant (**b**, **right**) across two different greenhouse rooms with variable temperature and humidity settings. Population densities were monitored weekly for a period of 10 weeks. Error bars are standard errors.

**Figure 4:**
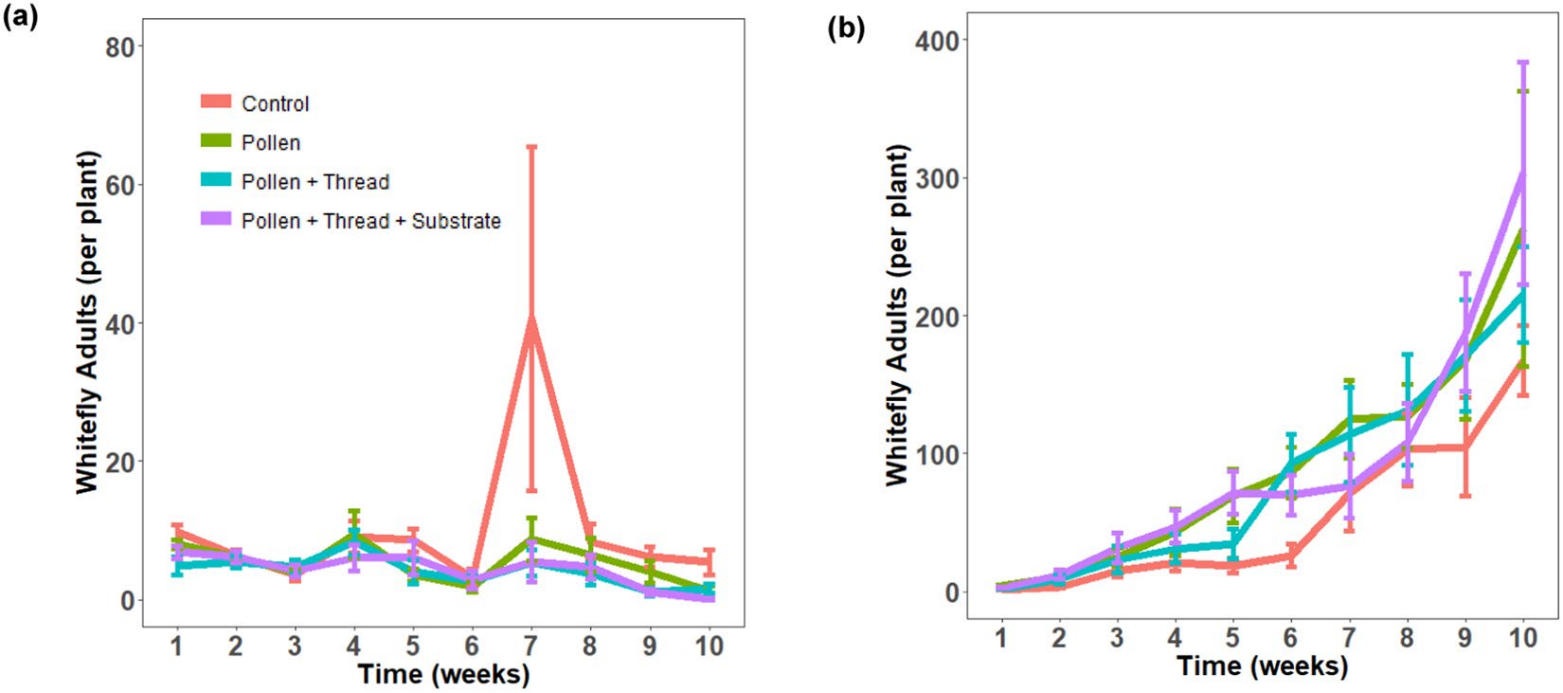
Comparison of the number of whitefly adults on two host plants. Effects of four different pollen, thread, and substrate treatments on adult greenhouse whitefly (*Trialeurodes vaporariorum*). This experiment utilised pepper (**a**) and eggplant (**b**) to assess differential treatment effects on different host plants. Experiments were conducted in two different greenhouse rooms with variable temperature and humidity settings where population densities were monitored weekly over 10 weeks. Error bars are standard errors.

Populations of whitefly eggs and juveniles exhibited a homogenous trend for both peppers and eggplants (Fig. 3). Very low population levels were detected until week 6 to week 7 where there was a dramatic increase for the control treatment in peppers and a dramatic increase for all treatments in eggplants. The control treatment had the highest population for both whitefly eggs and juveniles for pepper plants, but this effect was not significant for whitely eggs (Table 2). Contrastingly, no treatment effect was detected in eggplants with populations of whitefly eggs and juveniles increasing exponentially. For whitefly adults there was a weak treatment effect for peppers and no significant treatment effect for eggplants (Table 2). In the control treatment for peppers, there was a spike in whitefly adult populations in week 7 after the third release of whiteflies. However there was a general imperceptible decreasing trend throughout the experimental period and whitefly juveniles were essentially the main life stage that maintained at the conclusion of the experiment (Figs 3, 4). Adult whitefly populations on eggplants were discordant with that pattern, instead displaying a gradual increase across all treatments. Similar to the predatory mites, there was a huge disparity in whitefly populations between the two host plants with eggplants achieving a much higher population of whiteflies.

## Discussion

With the addition of pollen and/or substrates, significant control of greenhouse whitefly was achieved by *A*. *limonicus* on pepper plants but this was not observed on eggplants. Though no threshold level has been confirmed for greenhouse whitefly, it was defined at 5 juvenile/adult whitefly per leaf for this study. This level was utilised from a similar threshold value that has been established for *Bemisia tabaci* (sweet potato whitefly) which was defined at 4 adults per leaf^47^. By the end of the experiments, control was obtained by the three pollen treatments, but not in the treatment with no form of supplementation. For peppers, the provision of pollen and thread was one of the main factors determining predatory mite populations, and thus effectively contributed to efficient control of greenhouse whitefly. The addition of substrate material did achieve higher populations of *A*. *limonicus*, however, this was not significantly different from populations in the pollen and thread (PT) treatment.

The opposite trend was observed on eggplants with predatory mites unable to establish on eggplants while whitefly numbers increased exponentially after 2–3 weeks. All life stages of predatory mites were recorded at extremely low abundance while whitefly eggs and juveniles were recorded in the thousands. With no differential treatment effect being detected on eggplants, host plant appears to be the main determinant affecting both whitefly and mite populations.

### Trichome density and its impacts on *Amblydromalus limonicus*

**Figure 5:**
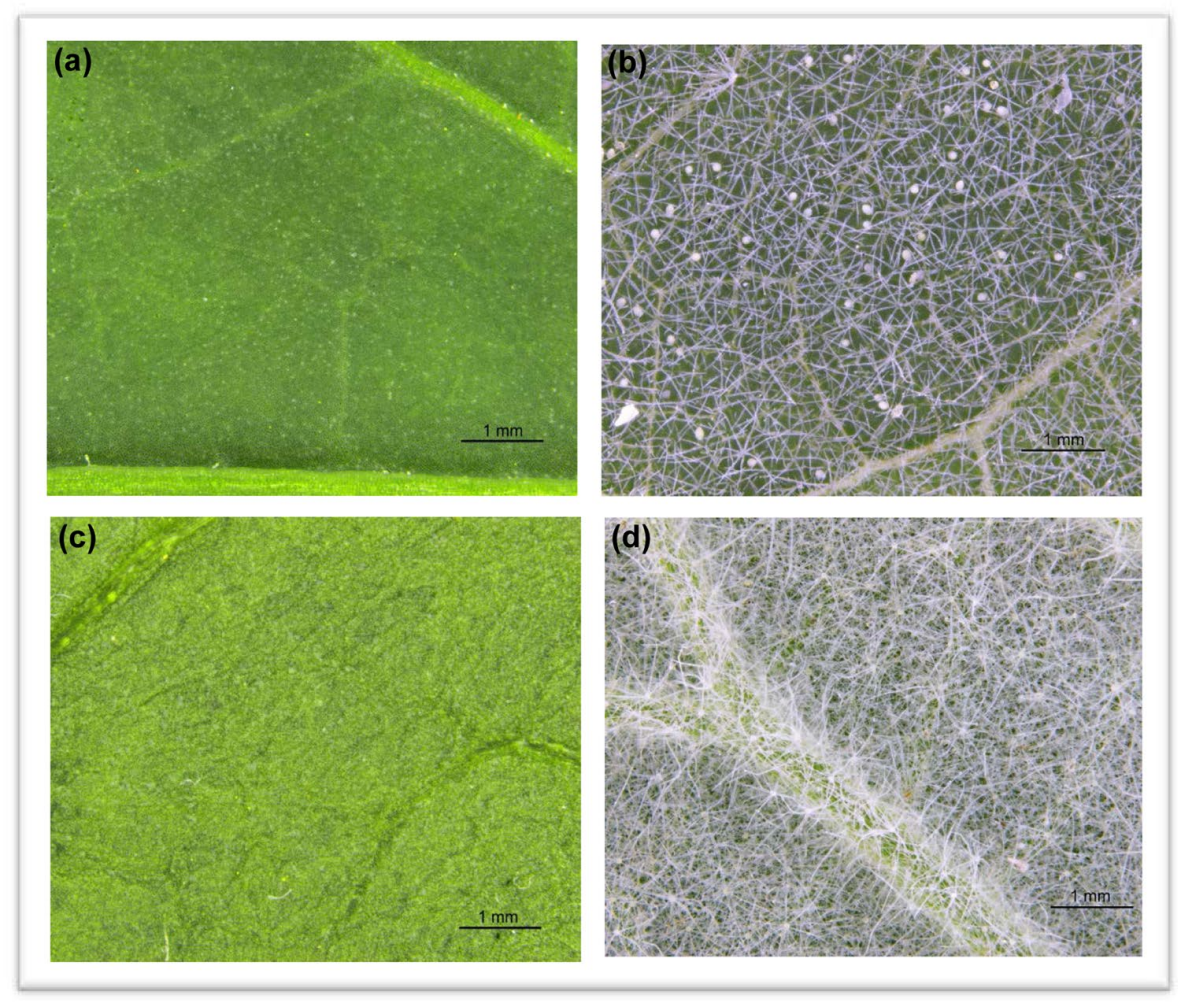
The two different host plants utilised to test the effects of four different pollen, thread, and substrate treatments on the ability of *Amblydromalus limonicus* to control greenhouse whitefly (*Trialeurodes vaporariorum*). The adaxial leaf surface comparisons of (**a**) pepper and (**b**) eggplant which highlight the different trichome densities of each plant. The abaxial surface of (**c**) pepper and (**d**) eggplant also display the same effect.

Plants have variable leaf structures which can both enhance and hamper the success of predatory mites^48^. Trichomes, for instance, act as a defence barrier against herbivorous arthropods, but may also be detrimental to natural enemy populations^49–53^. A recent review revealed that trichomes were more harmful than beneficial for arthropod predators^50^. These effects were mostly sub-lethal, reducing the movement, development, oviposition, and predation potential of beetles, true bugs and lacewings. In this study, the ability of *A*. *limonicus* to sustain a population on peppers was largely attributed to suitable leaf characteristics. The glabrous (non-trichome bearing) leaves present on pepper plants allowed the mites to navigate easily which effectively enhanced their ability to exploit all available food sources (Fig. 5). This also allowed predators to disperse when leaves became overpopulated and translated to better control of greenhouse whitefly. Contrastingly, for eggplants, where the leaves were covered with dense stellate trichomes that is characteristic of the Solanaceae family, *A*. *limonicus* were unable to establish^51,52^ (Fig. 5). The hairy leaves prohibited the movement of *A*. *limonicus*, which reduced the availability of oviposition sites and foraging ability^48,53–55^.

Epicuticular waxes and chemical exudates produced from the glandular trichomes on eggplant may have also prevented *A*. *limonicus* from exhibiting a strong numerical response^54,56,57^. The combination of these factors is likely to have caused the unsuccessful control of greenhouse whitefly on eggplants. In addition, the invasion of spider mites in week 6 may have also contributed to the decline of *A*. *limonicus*^25,32^. The predators have been shown to be adversely affected by webbing produced from spider mites with oviposition rates being drastically reduced. The combination of these factors is likely to have caused the unsuccessful control of greenhouse whitefly on eggplants.

Similar findings have been observed where predatory mites have been unsuccessful in establishing on plants with high trichome density. For example, the predatory mite, *Amblyseius swirskii* (Athias-Henriot), was also unable to build up a population on eggplants which led to ineffective control of western flower thrips (*Frankliniella occidentalis*, Pergande)^54^. The reduction of the foraging behaviour of *A*. *swirskii* was attributed to the dense stellate trichomes which led to the reduction of thrip predation. Congruently, an additional study showed that *A*. *limonicus* were not able to establish on tomato plants due to forests of glandular trichomes on leaf surfaces^31^. However, when these plants were compared to plants infested with the tomato russet mite (*Aculops lycopersici*, Tryon), predatory mites were able to establish in areas where high pest densities were found. Resultant feeding by the pest mite induced trichome collapse which allowed predatory mites to navigate easier and allowed for a sustained predator population. Additionally, *A*. *limonicus* females have been found to consume more psyllid nymphs when presented on pepper leaf discs compared to hairy tomato leaf discs^58^. These studies strongly support the conjecture that trichome density can act as a critical barrier to the establishment of predatory mites and hence their ability to act as a biological control agent.

Although there is literature that supports the findings of this study, the correlation between leaf trichomes and natural enemy populations are not consistent. In fact, most recent literature suggests a positive linear relationship between phytoseiid density and the presence of trichomes^48,59^. Trichome dense plants such as grapes and apples have been observed to have a larger abundance of the generalist phytoseiid *Typhlodromus pyri* (Scheuten) than cultivars with almost no trichomes^60–63^. In a dual choice test, *Phytoseiulus persimilis* (Athias-Henriot) showed a preference to reside and oviposit on surfaces with artificial trichomes composed of cotton fibers compared to glabrous lime bean surfaces^63^. Although this is advantageous in the rention of phytoseiids, a semi field test revealed that the walking speed of *P*. *persimilis* was lowest on daisy cultivars with high leaf hair density which resulted in lower search efficiency, and thus predation of two-spotted spider mites^49^. This increases the complexity of these interations and highlights the equivocal nature between trichome density, predatory populations, and control efficiency.

It has been suggested that non-glandular trichomes provide a structure which predators can use for pollen capture and protection from predation and cannibalism^48^. An increase in pollen capture can serve as an alternative food source for predators when prey are scarce. This was demonstrated when trichome-poor cultivars supplemented with pollen saw an increase in *T*. *pyri* populations, while populations on trichome-rich leaves did not^64^. The populations on trichome-poor cultivars rose to similar levels seen on the trichome-rich cultivar, indicating that pollen supplementation resulted in the trichome-poor leaves having similar pollen levels that were already present on the dense trichome cultivar. This relationship was not tested for in the present study due to the absence of flowering parts on host plants. Moreover, trichomes may provide refuge from intraguild predation and cannibalism. For example, thrips predation of *T*. *pyri* eggs on pubescent leaves collected from the field was significantly less compared to eggs consumed on glabrous leaves^65^. Trichomes can thus increase the habitat structure of a system which can facilitate better food capture, reduce encounter rates and decrease negative impacts of intraguild predation^45,48^.

These examples and the findings of the current study illustrate that host plant preference, in regards to trichome density, are highly variable and differ across species. A possible factor that dictates plant preference could be related to the level of specialisation seen in phytoseiids^66,67^. It has been suggested that specialists which have adapted to specific diets are less likely to be impacted by the host plant compared to generalists^48^. For example a study revealed that the genus *Neoseiulus*, which are largely composed of generalist predators, were highly host specific^68^. Each species of *Neoseiulus* sampled showed strong fidelity towards a particular species of plant with little to no overlap detected. Each of the species sampled are likely to have specific requirements such as distinct shelter or oviposition sites which are only satisfied by the particular host plant. However, it is essential to establish that although species within this genus have specific requirements, they are able to exploit variable prey within that specific microhabitat^68,69^. This supports the theory that generalists have a closer association with a host plant compared to specialist predators and may explain the drastic contrast of predator populations seen in the present study^48^.

### Greenhouse whitefly population dynamics and host plant preference

Host plants have been shown to have an effect on the oviposition rate, survivorship, immature mortality, and development time of greenhouse whitefly^6,42^. In a choice test, initial host plant choice was random however a clear preference was exhibited after 24 hours. Whiteflies show a clear preference for eggplants over cucumber, gerbera, lima bean, tomato, and peppers^42,70^. This same ranking was seen in regards to their performance which was a measure of their developmental time, survival rate, longevity, and fecundity^3,42,71^. This strong correlation between preference and performance is a probable contributing explanation for the differential population growth between the two host plants in the current study. Extremely high populations of all three life stages of whitefly were observed on eggplants, while whitefly populations struggled to establish on pepper plants. This effect on peppers was compounded by the successful establishment of *A*. *limonicus* which would have further reduced whitefly populations. In addition, due to the high whitefly population on eggplants, mass amounts of honeydew secretions were observed on both the abaxial and adaxial surfaces of the leaves. These leaves were soon colonised by black sooty mould fungi which further degraded the microhabitat for *A*. *limonicus*. As predators avoid leaves with a large population of whitefly, the area in which *A*. *limonicus* could habituate became increasingly restricted until they were no longer detected^72^.

### The role of pollen, twine, and substrate supplementation

Treatment effects were absent on eggplants presumably due to the poor host suitably for predatory mites and the high preference by greenhouse whitefly^42,58,73^. As the pollen and substrate treatments were only targeted towards increasing natural enemy persistence and establishment, no treatment effects were recorded on eggplant.

However for peppers, the pollen and thread (PT) and pollen, thread, and substrate (PTS) treatment yielded the highest mite populations on pepper plants while the control had the lowest. This effect was significant for mobile mite populations but not for mite eggs. A possible explanation for this could be due to the specific oviposition behaviours of *A*. *limonicus* which seek to reduce cannibalism by other individuals^72^. This may have led to the differential detection of eggs recorded in this study. In addition, after the experiments were terminated, the tea bag covers with substrates were opened and eggs and mobile mites were found hiding amongst the husks for the pepper plants. Though this finding was not consistent across all tea bags covers.

For peppers, the pollen treatment had significantly higher predator populations compared to the control. This is expected as predators could only utilise whitefly as a food source in the control, and *A*. *limonicus* has been shown to have long development times and low survival rates when consuming greenhouse whitefly^33^. Furthermore, no second generation of *A*. *limonicus* was obtained on the diet which solely consisted of greenhouse whitefly. This aspect does contrast what was found in the present study as populations of *A*. *limonicus* were still present at the end of the experiment on pepper plants. The presence of *A*. *limonicus* in the control treatment may be due to migration from other plants or the ability of the predator to utilise plant exudates^74^. It is possible but unlikely that spider mites and *P*. *persimilis* eggs contributed to the persistence of *A*. *limonicus* as they were both quickly eliminated on pepper plants. The low predator populations in the control treatment led to ineffective control with higher whitefly juvenile and adult populations recorded compared to other treatments.

The larger mite populations of the pollen treatment compared to the control is in accordance with what was hypothesised as pollen has been shown to be an effective additional food source for *A*. *limonicus*^24,75^. The significant difference between the pollen treatment and the PT and PTS treatment demonstrate the importance of additional oviposition sites on pepper plants. Mites were observed to oviposit extensively on the pieces of thread during the first two weeks of experiments, but eggs were rarely seen on thread thereafter. This absence was also seen even when there were large portions of thread that had not had been used. Predatory mites have been shown to be flexible in their oviposition behaviour, switching oviposition sites according to predation risk^70,76,77^. Due to the fact that *A*. *limonicus* are cannibalistic, predator cues which may trigger threat sensitive oviposition behaviour may have deterred gravid females from ovipositing in areas where other cannibalistic females have been. This anti-predation response may be a component of innate or learned behaviour where oviposition in areas with predator cues are avoided^76^. Regardless, the provision of thread allowed for a greater persistence of *A*. *limonicus* even after greenhouse whitefly populations were considered controlled. The predators also reached relatively high populations in the PT and PTS treatment considering only 10 mites were released on each plant.

Similar predator population size and levels of whitefly control between the PT and PTS treatment suggest that the addition of substrates did little to improve conditions. The substrates were predicted to provide additional oviposition and refuge sites thereby enhancing the establishment and persistence of *A*. *limonicus*^48,78^. However, this was not shown and a few hypotheses are suggested to explain this observation. Firstly, if any substrate effects were to occur, they would have been minimised as mould was found on the pollen and substrates in the tea bag covers. Although the growth of mould was not extensively throughout the substrates, the predators are likely to have preferred sheltering amongst leaves. Secondly, as mould developed on pollen in the tea bags covers, there was no available food source for newly emerging predators. There is evidence that the egg laying behaviour of predatory mites are highly linked to prey patch availability^79^. Lastly, the method used to supply substrates may have not been the most effective way of supplying substrates. As the substrate packs were placed on the lower half of the plant away from leaves, these refuge sites were not easily accessible. A more effective way of distributing these substrates could have allowed the husks to be utilised and perhaps produce a significant substrate effect.

### Practical advice for growers

Incorporating pollen and additional shelter will benefit predatory mite populations. This will assist with pest control and result in better return for greenhouse growers. In order to improve on the cost-effectiveness of biological control operations, this study has attempted to reduce the need for frequent reintroductions of predators. Crops with very hairy leaves such as eggplants and tomatoes may not be able to host predatory mites. Thus, it would be wise to limit the following recommendations to crops and ornamentals that are able to sustain predatory mite populations such as roses, peppers, and cucumbers^10^. The first and foremost recommendation would be to incorporate pollen as an additional food source. With *Typha* spp. pollen now being commercially available, growers no longer have to endure the labour intensive task of hand collecting pollen^33,36,39^. A “pollen gun” can also now be employed to quickly dust pollen over a large range of crops and ornamentals^36,80^. A light layer is sufficient to enhance predatory mite populations. Secondly, the incorporation of short pieces of thread is advised in greenhouses as many predatory females were observed to lay eggs on them. This correlated to a higher natural enemy population found on pepper plants with thread. Thread is inexpensive and was easy to prepare, so this may be a cost effective option to enhance natural enemy populations. The pollen gun would not be able to evenly distribute the thread around plants so they would need to be manually placed. It would also be helpful to include substrates into the system. When purchasing *A*. *limonicus*, the predator comes in small bottles mixed in with media. However, husks provide more space for the cannibalistic predators and thus can enhance their establishment. Though, it may be difficult to obtain all three husks used in this study, utilising just one type of husk is likely to have similar effects. Rice husk may be a good candidate as it is easier to obtain, and has been shown to impede mould growth compared to other husks^43^. As this is a new concept that requires more improvements and testing, no commercial production of such a product is available. Whilst this product would be relatively cheap to utilise, it will be more labour intensive. However, if a grower does want to proceed, it is suggested to trial husks in a tea bag on a few plants to assess its capability. Placing tea bags at different height levels may also be beneficial. These various applications may produce significant benefits for predator populations and hence have a positive impact on the output of agricultural and ornamental crops.

### Future recommendations

To fully establish the potential of *A*. *limonicus* as a biological control agent, a wider host plant variety needs to be assessed. Particularly, a separation based on trichome density with a further division between glandular and non-glandular trichomes would be a useful comparison. It has been found that up to a certain density of trichomes, trichome number has a negative effect on predator movement^53^. Moderate trichome densities have been shown to minimally affect predator walking speed while providing sheltered habitats^60,81^. The ability of trichomes to serve as shelter and oviposition sites can greatly enhance the efficacy of biological control and hence is worth further research^62,77^. Selective breeding or genetic engineering for crop plants that have the preferred attributes has been suggested and is a feasible option that could greatly contribute to pest suppression in the long term^48,81^. However, as whiteflies also show a preference for moderately dense trichomes, it is important to consider any possible ramifications that may develop amongst the complex trophic level interactions that commonly exist in a greenhouse system^50,81,82^.

## Conclusion

This is one of the few studies that have found a positive relationship between increasing phytoseiid densities and control of pests. A probable reason that contributed to effective control of greenhouse whitefly on pepper plants was the presence of *Amblydromalus limonicus*. The discrepancy in whitefly density between pepper and eggplants was likely due to a combination of predator and prey preference for each host plant. The glabrous leaves found on peppers were highly suited to *A*. *limonicus* while the dense trichome density of eggplants provided a suitable microclimate for greenhouse whitefly. However, these observations cannot be extended across the Phytoseiidae or Aleyrodidae family as host plant preference is hugely variable and differs across species. Significant treatment effects were found on pepper plants but this was not replicated across eggplants due to the inability of *A*. *limonicus* to navigate on the latter. The pollen-thread and pollen-thread-substrate treatments yielded the highest predator populations due to the supplementation of oviposition sites. The addition of substrates did not have a significant effect on *A*. *limonicus* populations. This study focused on the practical implementation and optimization of the use of predatory mites, which are now a cornerstone in integrated pest management practices. As more effective methods are being developed and discovered, more options are becoming available for agriculturalists. This allows growers to select the most appropriate species for use, and is thus a crucial step towards enhancing the utilisation of biological control.

## Methods

### Mite colony and plant cultures

*Amblydromalus limonicus* individuals were initially obtained from black nightshade (*S*. *nigrum*, Linnaeus) in St Johns, Auckland. Mites were reared on *Typha orientalis* (Presl) pollen that is similar to the pollen of the closely related *T*. *latifolia* species (Linnaeus). The latter pollen has produced the highest intrinsic rates of increase in *A*. *limonicus* compared to other pollen species^75^. The pollen used for the cultures and experiments was collected from the St Johns district in the summer of 2014. It was subsequently air dried using an oven at 30 °C and stored in a −18 °C freezer. Mite colonies were maintained on rearing trays within the laboratory facilities at Landcare Research, Auckland, New Zealand.

Rearing trays consisted of circular black plastic arenas (14.5 cm diameter) placed on an inverted Petri dish which were wrapped with three paper towels. Two rubber bands were used to fix the Petri dish to the plastic arena. This was then placed in a plastic tray containing distilled water to ensure that the tissue would remain wet and thus provide water access to the mites via the periphery of the Petri dish. Long pieces of thread were distributed across the tray to provide additional sites for refuge and oviposition. Supplementary food was provided by putting 10 three cm strips of cotton thread into a plastic vial containing pollen and shaken vigorously^39^. Excess pollen was then gently shaken off and the threads were spread around the plastic arena. The trays were then placed in a room with 12: 12 h (L:D) photoperiod at 25 °C ± 1 °C in 80% relative humidity. Pollen and water were supplemented every fifth day. Multiple trays were set up in order to maintain a large number of *A*. *limonicus*.

An estimated 500 adult greenhouse whitefly reared on tobacco plants (*Nicotiana tabacum*, Linnaeus) were purchased from Bioforce Limited (Auckland, New Zealand). These were reared in Landcare Research greenhouses using tomato and eggplants as host plants.

Twenty four eggplants were grown from seeds in plastic pots filled with Daltons potting mix (fertiliser, pine bark, and pumice). These were maintained in cages in a greenhouse at 25 °C with the natural day and night setting present from September to January in Auckland, New Zealand. The eggplants were grown for four months and were supplemented with Yates liquid fertiliser (fish emulsion with additional NPK 9:2:6 at 1% concentration) in the last month before experiments commenced. *Tetranychus urticae* (spider mites) were spotted in the second month which resulted in the release of 1000* P*. *persimilis* to eliminate the pest population.

In January 2016 extreme temperatures were recorded (>40 °C) and a reinvasion of spider mites occurred on the eggplants. Extreme webbing, stippling and senescence of leaves were observed. Over 13,000 *P*. *persimilis* were released over the course of a month in an attempt to overwhelm the spider mite population. After a month and a half from the first release, eradication of spider mites was achieved. Damaged leaves were removed and plants were trimmed to five new growth leaves.

Due to space limitations, 28 pepper plants were grown outdoors from seeds. Peppers have a much faster growth rate compared to eggplants so they were planted in late December and extracted from the ground at 12 weeks old. Plants were examined thoroughly for pests and the few leaves that contained thrips, aphids, and spider mites were removed before experiments began. No synthetic pesticides were utilised for either host plants due to its ability to confound the results. Seedlings were placed in the same plastic pots used for eggplants and trimmed to five leaves. The pepper plants were also supplemented with liquid fertiliser a month before experiments began to ensure plants were in optimal health.

### Experimental set-up

**Figure 6:**
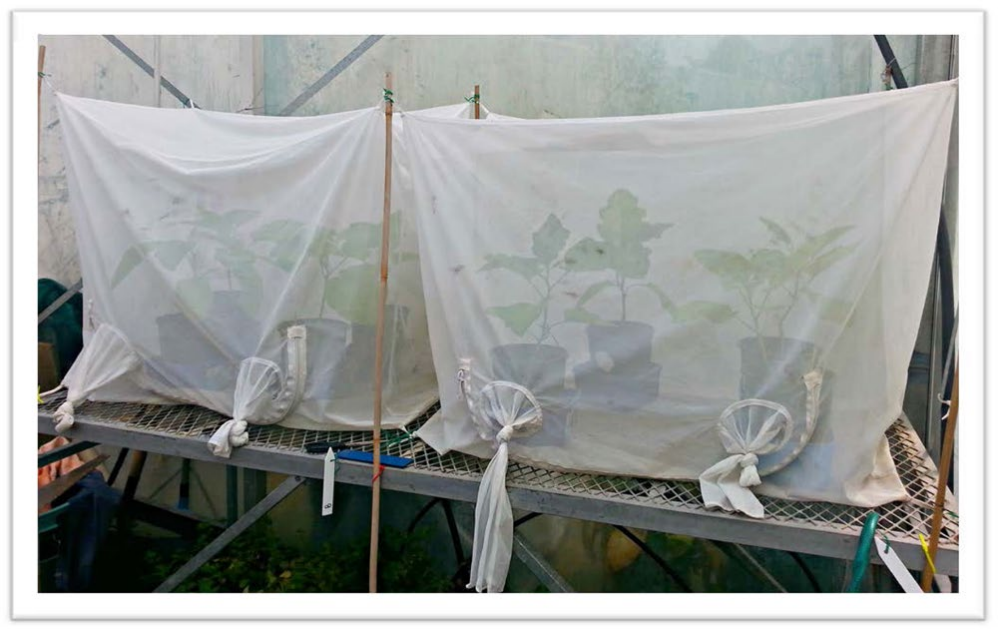
Cages with eggplants arranged in a 2 × 2 grid in the greenhouses located at Landcare Research, Auckland. This arrangement was used to assess how different treatments may affect the ability of *Amblydromalus limonicus* to control *Trialeurodes vaporariorum*.

The experiment was carried out in 13 cages (0.98 × 0.64 × 0.73 m), which were placed across 10 tables (Fig. 6). Due to space restrictions, pepper and eggplants were placed in two different greenhouse rooms. Additionally, as eggplants were recovering from spider mite damage, the experiments on pepper plants were initiated first with eggplants commencing 2 weeks thereafter. Both greenhouses had a photoperiod of 16:8 h (L:D) and variable temperature and humidity settings throughout the experimental period due to mechanical issues. Cages were handmade using fine nylon gauze (80 µm) which was maintained upright using metal framing and bamboo shoots. All cages were soaked in hot water with detergent for two days prior to being used for the experiment. Access was gained through a zip located in the front of the cage.

Due to limited space and number of cages, four plants, which represented each of the treatments, were placed in one cage. In this way, pseudoreplication was avoided for all mite stages and whitefly eggs and immatures. However, due to the ability of adult whitefly to migrate between plants within a cage, each treatment was not strictly independent, although they have been treated as such for statistical analyses. Plants with similar heights and leaf areas were grouped in fours and placed in a cage. The potted plants were then arranged in a 2 by 2 grid to ensure there was no contact between plants. Plants were irrigated once a week and supplemented with fertiliser fortnightly. Each plant was placed in a plastic tray consisting of a 2–3 cm layer of water. The plant was suspended above water using an inverted plastic lid which protruded out the water. An insect trap coating (Tanglefoot) was also spread around the edges of the water tray. In this way, both a water barrier and a physical barrier were employed to reduce the escape and invasion of mites and other pests. The experiment was set up so that as the plants grew, their leaves did not come in contact with the cage or other plants. Temperature loggers were placed in each cage to record the conditions at 15 minute intervals.

### Initial treatment conditions and pollen supply

**Figure 7:**
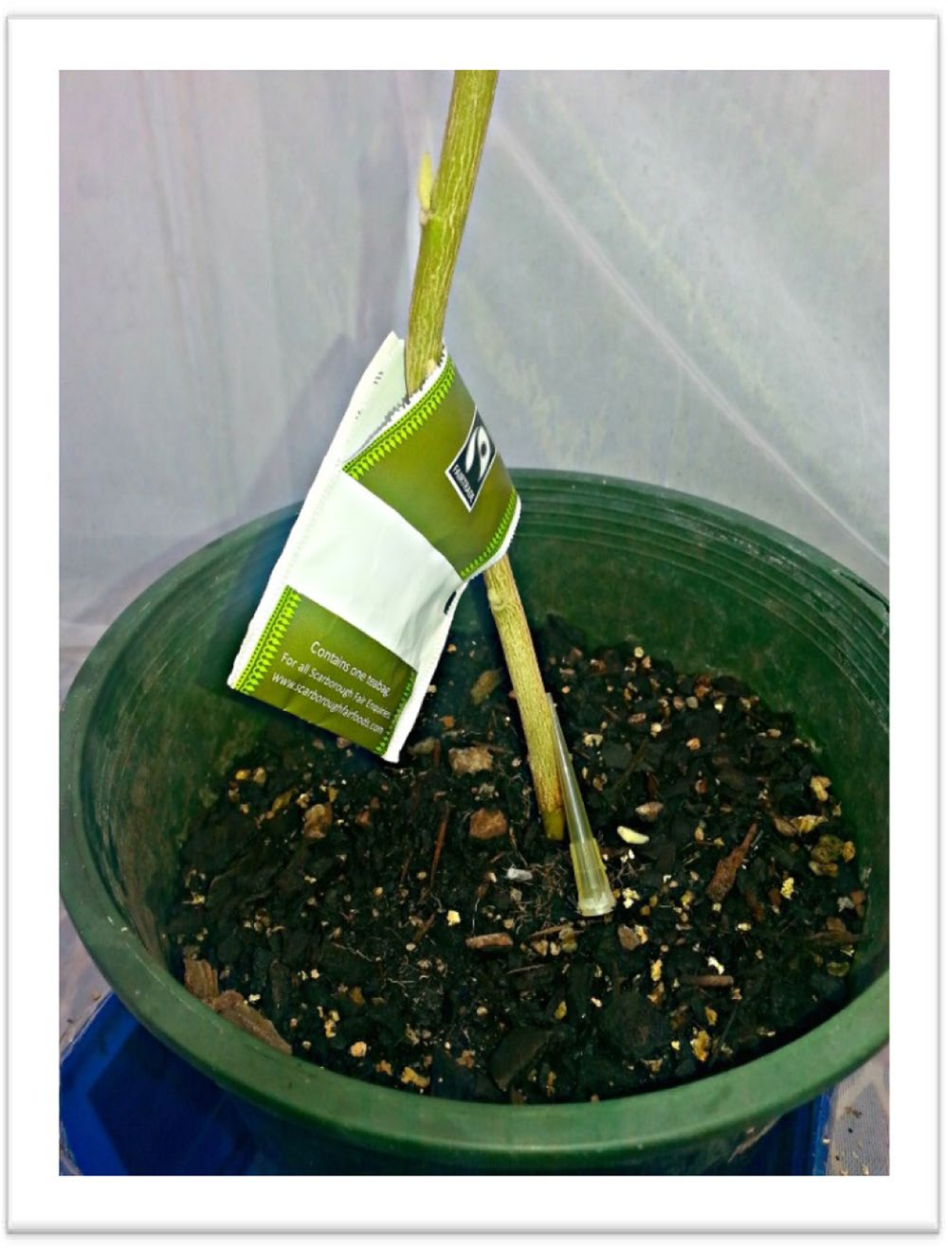
Substrates were placed in an opaque tea bag cover and hung on the plant stem. Pipette heads were used in the transfer of *Amblydromalus limonicus* to each host plant.

Sixty greenhouse whiteflies were collected using an aspirator and introduced into each cage. The plants were gently shaken each day prior to mite inoculation, to encourage an even distribution of whiteflies across each plant. This was followed by the release of 10 gravid female predatory mites per plant, five days after whitefly inoculation. Mites were gently aspirated and placed in a refrigerator at 11 °C to reduce their metabolic rate for 30 minutes while the other mites were being collected. The pipette heads were subsequently placed upright against the base of the stem of each plant (Fig. 7).

Each cage contained four different treatments which included: (1) a control treatment with no pollen, thread, or substrate, (2) pollen, (3) pollen and thread, and (4) pollen, thread, and a mix of buckwheat, gorse and rice husk. There were a total of 13 replicates for each treatment. The thread used in the experiment was made from cotton and had a major diameter of 0.4 mm. Two pieces of 15 cm thread was hung on the lowest two leaves and the mix of husks were placed in an opaque green tea bag cover and placed onto the stem horizontally (Fig. 7). As populations of predatory mites have been observed to benefit from pollen coated on leaves rather than twine, this method was applied^39^. Plants that received pollen were supplied with 4–6 mg of pollen split between two leaves, with the exception of the treatment which included husks, where only half the amount of pollen was spread between the two leaves and the other half was spread into the tea bag cover. Pollen treatments were supplemented with new pollen weekly as pollen has been demonstrated to remain fresh for at least a week^83^.

#### Monitoring population dynamics

Assessment of whitefly and predatory mite populations on the abaxial surface of each leaf were done *in situ* using a 20x hand lens magnifier. Life stages were classified into mite eggs, mobile mites (nymphal and adult stages), whitefly eggs, whitefly juveniles (nymphal and pupal stages), and whitefly adults. When whitefly eggs and juveniles were so high that accurate counts could not be conducted, a 1 cm^2^ area was sampled and extrapolated to the entire leaf. Sampling on pepper plants and eggplants were undertaken on different days to reduce the likelihood of indirect transfer across different host plants. This sampling regime was initiated five days after predator release and was run for a total of 10 weeks with the prey and predator population dynamics evaluated weekly. In addition, the stem diameter of each plant was measured using a calliper, on the same day the mites were released, and then again at the conclusion of the experiments.

During the first week of monitoring, 10 adult spider mites were spotted on two pepper plants so five *P*. *persimilis* were released on each pepper plant across all cages. *Phytoseiulus persimilis* is a specialist predator of spider mites and is thus unlikely to have affected *A*. *limonicus* or whitefly populations^67,84^. Correspondingly, during week 6 of monitoring on eggplants, spider mites were increasingly seen which resulted in the release of 1000 *P*. *persimilis* across all cages containing eggplant. Additionally, low whitefly populations recorded on peppers resulted in an additional 60 adult whiteflies released per cage in week 4 and another 100 during week 6 for both host plants.

#### Statistical analyses

A repeated measures analysis of variance (ANOVA) was applied for mite eggs, mobile mites, whitefly eggs, whitefly juveniles, and whitefly adults over the 10 week period for each host plant. Treatment was incorporated as a factor while the week number was added as an error term. Log transformations were applied for data that violated normality or variance assumptions (+1 was added to all y values to eliminate zeros). Additional pairwise comparisons were conducted for results that were shown to be significant.

An ANOVA was used to assess any differences in temperatures between the different greenhouse rooms. Similarly, a multistrata ANOVA was applied to examine any differences in the change in stem diameter between treatments and different host plants. All statistical analyses were done using the R Foundation for Statistical Computing package (version 3.3.1).
